# The women’s heart health programme: a pilot trial of sex-specific cardiovascular management

**DOI:** 10.1186/s12905-018-0548-6

**Published:** 2018-04-16

**Authors:** Ting Ting Low, Siew Pang Chan, Shin Hnin Wai, Zhou Ang, Kyu Kyu, Kim Yee Lee, Anne Ching, Sarah Comer, Naomi Qiu Pin Tan, Elizabeth Grace Hui En Thong, Tracy Nang, Mohan Dutta, Carolyn S. P. Lam

**Affiliations:** 1 National University Heart Centre, Singapore, Singapore; 2 National University of Singapore, Singapore, Singapore; 3 Monash University, Clayton, Australia; 4 Imperial College, London, UK; 5 New York University, New York, USA; 6 National Heart Centre Singapore, 5 Hospital Drive, Singapore, 169609 Singapore; 7 Duke-National University of Singapore, Singapore, Singapore

**Keywords:** Women’s health, Healthcare disparities, Cardiovascular disease, Quality of life, Health knowledge, Attitudes, Practice, Culturally competent care

## Abstract

**Background:**

There is increasing knowledge of sex-specific differences in cardiovascular disease and recognition of sex disparities in management. In our study, we investigated whether a cardiovascular programme tailored to the specific needs of women could lead to improved outcomes.

**Methods:**

We randomised 100 female patients to receive cardiology follow-up with the conventional sex-neutral cardiac programme (control), or the sex-tailored Women’s Heart Health Programme (intervention). The intervention group was managed by an all-women multidisciplinary team and received culture-centred health intervention workshops, designed through in-depth interviews with the participants. The primary outcome was cardiovascular risk factor improvement at 1 year. Secondary outcomes include cardiovascular event rates, quality of life scores, and self-reported improvement in knowledge, attitudes, intentions and practices. Generalised structural equation model analysis was used to determine if the intervention group had better outcomes at alpha level 0.1.

**Results:**

The mean age was 67.3 ± 12.7 years, with an ethnic distribution of 70% Chinese, 18% Malays, and 12% Indians. The majority of these patients had no formal or primary level of education (63%), and were mostly unemployed (78%). Patients in intervention group had better control of diabetes mellitus (lower HbA1c of 0.63% [CI 0.21-1.04], *p* = 0.015) and lower body-mass-index (0.74 kg/m^2^ [CI 0.02-1.46], *p* = 0.092) at 1 year, but there was no significant difference in blood pressure or lipid control. Overall, there was a trend towards better risk factor control, 31.6% of intervention group versus 26.5% of control group achieved improvement in at least 1 CV risk factor control to target range. There was no significant difference in incidence of cardiovascular events, quality of life, or domains in knowledge, attitudes, intention and practices.

**Conclusion:**

This pilot study is the first of its kind evaluating a new model of care for women with heart disease. The potential to improve outcomes needs to be studied in a larger trial with longer follow up.

**Trial registration:**

This trial was prospectively registered clinicaltrials.gov on 6 May 2013. Trial Number: 2013/00088. Identifier: NCT02017470

## Background

Over the past two decades, there has been a wealth of data illustrating sex differences in cardiovascular (CV) disease. Yet, there has been paucity of data examining how we can change the way we manage or try to alter any sex-specific patient outcomes.

Sexual dimorphism in CV disease extend across a huge spectrum [1]. First, there are pronounced sex differences in clinical phenotypes. For example, in heart failure syndromes**,** women tend to get heart failure with preserved ejection fraction whereas men get heart failure with reduced ejection fraction [2]. Some CV conditions also have a greater propensity to affect women more than men, such as apical ballooning syndrome and pulmonary arterial hypertension. There are also sex differences in pathophysiology and symptomology even for the same condition. For example, compared to men, women with ischemic heart disease may have microvascular disease instead of large epicardial coronary disease [3]. Atherosclerosis of the coronary arteries in women may be associated with extrinsic remodeling, such that disease is not always detectable as narrowing on usual coronary angiography. Rather than typical chest pain, women are more likely than men to experience atypical symptoms of angina, such as breathlessness, fatigue, nausea, indigestion and neck and jaw pain. Beyond this, there are sex differences in sensitivity with regard to multimodality imaging in the detection of coronary ischemia [4]. Finally, women are also known to have altered risks for the same condition such as increased stroke risk for female sex in the CHA_2_DS_2_-VASc score used in atrial fibrillation [5].

Despite emerging knowledge on sex differences, CV disease remains the most prevalent cause of morbidity and mortality among women. Alarmingly, even as there is an overall decline in CV mortality in both men and women, CV mortality amongst young women between ages 35-54 is increasing [6]. In terms of hospitalisation, most hospitalised for coronary heart disease above 65 years of age are women, and women constituted 74% of hospital stays for non-specific chest pain among patients above 85 years of age, higher than any other condition examined [7].

In addressing this burden of disease on women, dedicated heart clinics for women have been sprouting in many world renowned tertiary cardiac centres in recent years. These clinics offer a unique practice, designed with a sex-specific approach to focus on women’s CV health. Often, these clinics provide subspecialty management of CV conditions that affect women more than men, and also have a special focus on risk management, lifestyle modification and prevention of heart disease. Additionally, the women heart clinics serve an educational component, raising public awareness and highlighting importance of heart disease in women. It is also not uncommon that these clinics are run by an all women healthcare team, with the aim of providing better patient-provider communication, having women advocating for each other.

To date, there has been no studies evaluating the effectiveness of a dedicated women’s heart health (WHH) programme. We hypothesize that a dedicated CV programme, based on sound knowledge of sex differences in CV disease and tailored to the specific needs of women, may help close the treatment gap in women and improve their clinical outcome.

### Aim

The aim of this study is to compare the clinical outcomes (CV risks factor control and CV event rates), quality of life and the self-reported knowledge, attitudes, intentions and practices amongst women already known to have CV disease, when managed in 2 outpatient treatment groups at our institution: 1) conventional sex-neutral cardiac programme (control), and 2) sex-tailored women’s heart health programme (intervention).

## Methods

### Patient Population & Selection

A total of 100 female patients were recruited consecutively from the outpatient general cardiology clinics. The patients were approached for participation in the study while waiting to see their cardiologists in the clinic. All female patients aged 21-99 years old, diagnosed with any CV condition that required regular follow-up in the subsidised general cardiology clinic were included. Patients on follow-up in cardiac sub-specialty clinics such as the arrhythmia clinic, adult congenital heart clinic, cardiomyopathy clinic and valve clinic were excluded, as these patients often had more complex cardiac conditions that needed a different care model. We excluded patients who were unable to answer questionnaires and were not likely to require follow up with a cardiologist beyond 1 year.

Randomisation to the intervention and control groups was performed by a computer generated code, which assigned each participant to be followed up either in the usual general cardiology clinic (control group) or the women’s heart clinic (intervention group).

### The Women’s heart health Programme

The women’s heart programme was helmed by a multidisciplinary all women’s team (consisting of cardiologist, physiotherapist, occupational therapist, dietician and cardiac rehabilitation nurse) in a conducive environment for optimal patient-provider communication. In the intervention group, the patients were also engaged to develop heart health promotion strategies rooted in patients’ culture. This was done in collaboration with the Department of Communications and New Media, Faculty of Arts and Social Sciences from the National University of Singapore. Patients in the intervention group were interviewed to understand their preferences for women’s heart health intervention strategies, and invited to participate in facilitated focus group discussions. Eventually, a woman’s heart health promotion workshop was borne out of these conversations, which comprised of combined interactive sessions with the physiotherapist, occupational therapist, and dietician. The half-day workshop was conducted separately in 3 different languages (English, Chinese and Malay) and patients could choose to attend the one in their preferred language. In the control group, the patients may also be referred to the physiotherapist, occupational therapist and dietician by their managing cardiologist on an ad-hoc basis, without the combined structured program as in the workshop. The appropriate referrals are left to the discretion of the managing cardiologist.

### Blinding

While the cardiologists in the study were blinded to the patient’s assignment, we were unable to blind our participants to their grouping. In the control group, the patients may have a male or female cardiologist for review every 4 months, whereas in the intervention group, the patients consistently saw only female doctors each time. The female patients assigned to the intervention group also received more interviews in order to develop an integrated health intervention programme.

### Primary and secondary outcomes

The primary outcome measured include a change in CV risk factor control at 1 year from baseline. These include body mass index (BMI), systolic blood pressure (SBP), high density lipoprotein (HDL), low density lipoprotein (LDL), and glycated haemoglobin (HbA1c). The secondary outcomes measured include a) CV event and mortality rates, b) quality of life indices (i.e., mental and physical health scores constructed with the MOS SF-36), and c) self-reported results of knowledge, attitudes, intention and practices towards women’s health in Singapore, while adjusting for all relevant baseline factors.

### Follow-up

In both control (sex-neutral programme) and intervention (sex-tailored programme) groups, the patients were followed up equally at 4 monthly intervals for a duration of 1 year. The outcomes of CV risk markers (BMI, SBP, LDL, HDL, Hba1C) were assessed in clinic at baseline upon study entry and at the end of 1 year follow-up. A CV event was defined as an admission for myocardial infarction or stroke or CV death. The participants were asked if they had any CV events, emergency room visits or hospitalizations at each follow up. They were also instructed to inform our study coordinator if there was such an event. The hospitalisation records were made accessible to the study coordinator, and diagnoses were extracted from these records. The severity of comorbid conditions was summarized in the Charlson score with 10-year probability of survival [8, 9]. The MOS SF-36 physical component score (PCS) and mental component score (MCS) were constructed from the eight subscales of general health, health change, emotional well-being, pain, energy/fatigue, role functioning (emotional), role functioning (physical) and social functioning. A heart health survey on knowledge, attitudes, intention and practices was administered to all patients in the study at baseline and at the end of 1 year.

### Statistical analysis

The continuous variables were expressed as mean ± standard deviation or median/interquartile range, while the categorical variables were presented as frequencies and percentages. Exploratory analyses were carried out with Chi square test, Fisher’s exact test, independent *t*-test and Wilcoxon-Mann-Whitney test, depending on the nature (parametric vs. nonparametric) of data. The confirmatory analysis was carried out with the generalized Structural Equation Model (gSEM) [10, 11] to ascertain if there was a significant intervention effect on the clinical outcomes, self-assessed Quality of Life and self-reported knowledge, attitudes, intentions and practice outlined above, while adjusting for baseline factors (i.e. Charlson survival probability, CV risks, self-assessed quality of life and marital status: married/not married). Analysed with STATA MP Version 14.0 (STATA Corp, Texas, USA), all statistical tests were carried out at alpha level 0.1. We used alpha level 0.1 as an acceptable level of posteriori instead of the conventional alpha 0.05 to define statistical significance, so as to generate more meaningful results in an insightful way from this small descriptive study.

## Results

We consecutively recruited 100 female participants from the outpatient general cardiology clinic. We approached 132 patients who were eligible to participate but 32 women declined to be enrolled for unknown reasons. Ten patients were lost to follow up at 1 year visit, of which 8 are from the control group. The women’s heart health promotion workshop, which was provided free to the participants, only had a 40% attendance rate. Many cited reasons such as lack of available timing, lack of transport to hospital or even lack of interest in therapeutic lifestyle change.

### Baseline characteristics

The mean age was 67.3 ± 12.7 years. In this multi-racial study population, there were 70% Chinese, 18% Malays, and 12% Indians. Demographic characteristics were similar between the control and intervention groups (Table 1). The majority of these patients had no formal or primary level of education (63%), and were mostly unemployed (78%).

**Table 1:** Baseline characteristics: demographics and co-morbid conditions

	Control (*n* = 50)	Intervention (*n* = 50)	*p*-value
Age	67.3 ± 13.9	67.3 ± 11.4	0.988
Ethnicity			
Chinese	32 (64%)	38 (76%)	0.420
Malay	11 (22%)	7 (14%)	
Indian	7 (14%)	5 (10%)	
Marital Status			
Not Married	14 (28%)	10 (20%)	0.349
Married	36 (72%)	40 (80%)	
Education			
Primary & Below	32 (64%)	31 (62%)	0.759
Secondary	15 (30%)	14 (28%)	
Tertiary	3 (6%)	5 (10%)	
Smoking Status			
Non-Smoker	47 (94%)	47 (94%)	0.549
Ex-Smoker	0 (0%)	1 (2%)	
Current Smoker	3 (6%)	2 (4%)	
Comorbidities			
Hypertension	41 (82%)	43 (86%)	0.585
Hyperlipidemia	37 (74%)	36 (72%)	0.822
Diabetes Mellitus	22 (44%)	20 (40%)	0.685
Atrial Fibrillation	17 (34%)	12 (24%)	0.271
Heart Failure	4 (8%)	6 (12%)	0.494
Ischaemic Heart Disease	29 (58%)	32 (64%)	0.539
Prior Stroke	5 (10%)	8 (16%)	0.419
Chronic Kidney Disease	5 (10%)	10 (20%)	0.161
Peripheral Vascular Disease	6 (12%)	4 (8%)	0.505
Charlson 10-Year Survival Probability	0.39 ± 0.34	0.37 ± 0.33	0.766

The spectrum of CV disease and related conditions were also similar between the control and intervention groups (Table 1). The majority had hypertension (84%) and hyperlipidaemia (73%), while 42% had diabetes and 38% had all three conditions. The most common cardiac condition was ischemic heart disease (IHD), affecting 58% and 64% of patients in the control and intervention group respectively. The next most common cardiac condition was atrial fibrillation (AF), accounting for 34% and 24% of patients in the control and intervention groups respectively. Very few of the patients were current (1%) or ex-smokers (5%). The control and intervention groups were also similar in terms of their estimated Charlson probabilities of 10-year survival (Table 1).

### Cardiovascular risk markers

The median and interquartile range values of the CV risk markers (i.e. BMI, SBP, HbA1c, HDL and LDL) at baseline and 1 year are depicted in Table 2. While there was no significant difference between the control and intervention groups according to the bivariate analysis, on confirmatory analysis after adjusting for baseline difference, the intervention group had a significantly lower average HbA1c by 0.63% and a lower average BMI by 0.4 kg/m^2^ at the end of 1 year (Table 3). Separately, using cut-off targets for each of the 5 measured CV risk factor (goals of BMI < 25 kg/m2, SBP < 140 mmHg, HDL > 1.3 mmol/L, LDL < 2.6 mmol/L and Hba1C < 7%), we found that intervention was associated in endpoint changes that trended in the direction of improvement. Comparatively, 31.6% of intervention group versus 26.5% of control group achieved improvement in at least 1 CV risk factor control to target range, but this difference was not significant (*P* = 0.31). This was after multivariable adjustment for baseline factors including age, race, Charlson survival probability, CV risks, self-assessed quality of life and marital status.

**Table 2:** Cardiovascular risk factor control and quality of life indices at baseline and 1 year

	Control (*n* = 50)	Intervention (*n* = 50)	*p*-value
Body-mass-index (kg/m^2^)			
Baseline	26.1 (23.0-29.6)	25.7 (23.3-28.3)	0.539
1-Year	25.2 (22.5-28.8)	23.7 (22.5-26.9)	0.318
Systolic Blood Pressure (mmHg)			
Baseline	138 (120-150)	140.5 (130-153)	0.292
1-Year	137 (123-154)	140.0 (127-153)	0.702
Glycated Haemoglobin, Hba1C (%)			
Baseline	6.2 (5.6-6.7)	6.3 (5.7-7.4)	0.263
1-Year	6.3 (5.6-8.2)	6.2 (5.7-6.7)	0.202
Low Density Lipoprotein (mmol/L)			
Baseline	2.5 (1.9-3.0)	2.2 (1.9-3.0)	0.712
1-Year	2.3 (1.8-2.7)	2.4 (1.9-2.9)	0.465
High Density Lipoprotein (mmol/L)			
Baseline	1.3 (1.1-1.5)	1.3 (1.1-1.6)	0.344
1-Year	1.2 (1.0-1.5)	1.3 (1.1-1.5)	0.369
MOS SF-36 Scores			
Baseline			
Physical Component Score	51.9 (38.1-67.5)	61.3 (44.4-78.1)	0.077 *
Mental Component Score	54.6 (31.5-81.3)	79.3 (56.0-86.3)	0.014 *
1-Year			
Physical Component Score	71.3 (48.8-83.1)	77.5 (57.5-86.3)	0.160
Mental Component Score	79.4 (62.1-88.0)	81.0 (65.0-92.0)	0.613
Admissions (Cardiovascular)	8 (16%)	8 (16%)	0.999
All-Cause Mortality Status	2 (4%)	2 (4%)	0.999
Cardiovascular Event	5 (10%)	4 (8%)	0.727

*Statistically significant at 10%

**Table 3:** Analysis of covariance of clinical outcomes at 1 year after adjusting for baseline (Intervention vs. Control)

Outcomes at 1 year	Coefficient	90% C.I.	*p*-value
Body-mass-index (kg/m^2^)	−0.4	−1.46 — -0.02	0.092 *
Systolic Blood Pressure (mmHg)	−1.58	−11.44 — 8.29	0.791
Glycated Haemoglobin, Hba1C (%)	−0.63	−1.04 — -0.21	0.015 *
High Density Lipoprotein (mmol/L)	0.04	−0.11 — 0.18	0.680
Low Density Lipoprotein (mmol/L)	0.10	−0.14 — 0.35	0.486
Cardiovascular Event (Adjusted Odds Ratio)	0.78	0.20 — 3.10	0.727

*Statistically significant at 10%

### Cardiovascular clinical events

A total of 16 patients (control: 8, intervention: 8) were hospitalised for cardiac causes during the follow up period (Table 4). There was no significant difference in the frequency of cardiac admissions between groups (control: median 1.5, range 1-3; intervention: median 2, range 1-4). These comprised of admissions through the emergency department, and the diagnoses included acute coronary syndromes, decompensated heart failure, hypertensive urgency, angina and arrhythmia.

**Table 4:** Hospitalisations and major adverse cardiovascular events in both groups

	Control n (%)	Intervention n (%)	*p*-value
No. of patients admitted for cardiac causes	8 (16)	8 (16)	0.999
Frequency of cardiac-related admissions	13 (26)	16 (32)	0.383
Frequency of major adverse cardiovascular events	5 (10)	5 (10)	
Myocardial infarction	4 (8)	2 (4)	0.678
Stroke	0 (0)	2 (4)	0.495
Cardiovascular death	1 (2)	1 (4)	0.999

A total of 10 major adverse CV events were reported (control: 5, intervention: 5) in 9 patients (control: 5, intervention: 4). These included myocardial infarction (control: 4, intervention: 2), stroke (control: 0, intervention: 2) and CV deaths (control: 1, intervention: 1).

### Quality of life

There was no significant difference in the self-assessed physical and mental health between the two groups according to the same model estimated with gSEM, although there was some evidence suggesting that the intervention group had a better assessment in their physical health at baseline and at 1 year. Patients who had a higher HDL and a better 10-year survival profile reported a higher score for their physical health. It was interesting to note that married patients reported a significantly higher score for their mental health, other things being equal.

### Knowledge, attitudes, intention and practices

The results of the self-reported Heart Health Survey concerning patients’ knowledge, attitudes, intentions and practices were analysed separately within the same model. A detailed process of factor identification process was performed to ascertain which survey questions were loaded on the knowledge, attitudes, intentions and practice scores. It was confirmed that only 8 out of 26 questions in the knowledge domain were significantly loaded on a common knowledge score and all these were related to patients’ knowledge of the impact of cholesterol and blood pressure on their heart health. For the attitudes domain, all 3 questions (heart health risk for women, effectiveness of different kinds of medications and procedures for heart disease and prevention information for heart disease) were loaded significantly on one general attitudes score. While the same was observed for intentions (4 questions) there was no unique factor identified for practice (7 questions). The patients interviewed were not homogenous in terms of their reported practices for their heart health. It was further confirmed with gSEM that there was no significant group difference in the four domains.

## Discussion

To our knowledge, there had been no prior randomised study evaluating the benefits of a women-specific CV management approach by direct comparison to a conventional sex-neutral programme. This pilot study provides ‘real-world’ insights into how a tailored CV programme may benefit women, especially in a multicultural South-East Asian population as ours.

Our clinical trial is distinct for a few reasons. First, our study population consisted of a unique melange of ethnic and cultural diversity, with 3 main racial groups and multiple native languages. Not all participants were fluent with English language as a medium of communication, the women also spoke Chinese, or Malay or other forms of dialects. Using a novel cultured-centred approach, we explored the benefits of engaging the women in the creation of health promotion messaging and intervention implementation complementary to clinical measures.

The effectiveness of our women heart health programme was measured by both quantitative and qualitative analysis. In our study, the primary outcome was met for diabetes and BMI. Women who were managed in a sex-tailored heart health programme for a year, achieved a significantly better improvement in diabetes control and weight loss. Overall, there were more women in the sex-tailored programme who achieved at least 1 CV risk factor control to target range, compared to women in the sex-neutral programme.

The secondary outcomes were not met in this study. The implementation of sex-tailored heart health programme did not result in a significant change in quality of life, hospitalisation rates and survival at the end of 1 year. The women also fared similarly in terms of knowledge, attitude, intention and practices with regards to understanding their CV risks and lifestyle modification.

Our study participants were regular follow up patients recruited from the outpatient cardiology clinics. It was likely that a change in model of care did not significantly affect the secondary outcomes because our participants have cardiac conditions that were already well-stabilised in the ambulatory setting. Nonetheless, it is worth highlighting that both groups of women reported an improvement in quality of life from baseline, regardless of whether they were in the sex-tailored or conventional sex-neutral programme.

The women in the sex-tailored programme had a significant reduction in HbA1C by 0.6% at the end of 1 year. This improvement was also consistent with the trend towards greater weight loss in the intervention group. Based on American Diabetic Association and National Institute for Health and Clinical Excellence treatment guidelines, 0.5% HbA1c is considered a clinically significant change [12, 13]. Women with diabetes were found to have more than a 40% greater risk of incident coronary heart disease compared with men with diabetes [14]. Among people with diabetes, the prognosis of heart disease was shown to be worse for women than for men; myocardial infarction occurred earlier in women with diabetes compared with men [15], with higher mortality from the myocardial infarction [16]. In a nationwide prospective cohort study of 13,389 survivors post myocardial infarction, significant two-factor interactions were observed between sex, age and diabetes (*p* < 0.001). Diabetic women below 60 years of age had greater mortality than diabetic men of the same age (adjusted hazard ratio 1.44, *p* = 0.003) [17]. Although the mechanism underlying this excessive coronary artery disease risk in diabetes has not been fully elucidated, several hypotheses suggest that diabetes per se may be a stronger coronary risk factor in the female sex, determining a more unfavourable coronary risk profile [18, 19]. The excess risk of stroke associated with diabetes was also previously demonstrated to be higher in women than men [20]. These sex differences in CV consequences of diabetes is a call for more aggressive treatment strategies tailored for women.

There was equal improvement in BP, HDL and LDL targets whether the women were in a sex-tailored or sex-neutral programme. This could be explained by the already good blood pressure and lipid control at baseline in both groups, making it hard to demonstrate the effectiveness of our intervention programme. According to the National Health and Nutrition Examination Survey, women with hypertension are less likely to achieve blood pressure control compared to men [21]. Also, studies have revealed that women are less likely to achieve recommended lipid goals or to be receive lipid-lowering therapies compared to men [22–24]. Although we did not have sex-matched controls in this study, the overall good CV risk factor control at baseline suggest that sex disparity in BP and lipid treatment goals is unlikely in our institution.

There was a sizable proportion of women with AF, accounting for 29% prevalence in our cohort. Generally, females are more symptomatic compared to males for AF, with a higher proportion being in European heart rhythm association Class III and IV [25]. Women suffering from AF show a different prognosis, with a higher incidence of stroke and a higher mortality rate with respect to men [26, 27]. The Framingham Heart Study found that patients with AF showed a risk-factor-adjusted odds ratio (OR) for death of 1.5 in men and 1.9 in women [28]. This underscores the importance of engaging women to achieve aggressive CV risk factor modification, above and beyond standard medication for rate/rhythm control and anticoagulation.

The culture-centred approach was undertaken because it was illustrated previously that the cultural context and lived experiences of women are missing from programs and policies related to heart health in Singapore [29]. Major lifestyle changes are often difficult to institute or maintain especially when health messages are imposed from a “top-down” method but research has shown that women are better able to identify with heart health solutions if these solutions are presented in a way that is meaningful to them. In addition, a prospective nationwide study of 15,151 patients across 12 years also revealed that sex differences in CV death varied by ethnicity [30]. For example, compared to Chinese women, Malay women had the greatest increased hazard of CV death after an acute myocardial infarction (hazard ratio 1.4, 95% CI 1.2-1.6). We acknowledged this interplay of ethnicity and cultural attitudes on CV outcomes, and therefore took measures to accommodate these differences in secondary prevention strategy.

Unfortunately, despite a sex-tailored, culture-centred heart health workshop that was free of cost and conducted in various languages catering to different ethnicities, participation rate was poorer than expected (attendance rate 40%). Many cited reasons such as lack of available timing, lack of transport to hospital or even lack of interest in therapeutic lifestyle change. This highlighted the social barriers in effecting lifestyle modification for our female patients. Previous literature have shown that that while women are able to identify common CV disease risk factors, they often do not personalize this information, in other words, they do not perceive themselves to be at risk even when they have multiple risk factors [31]. This lack of perceived personal susceptibility can hinder prevention-seeking behaviour.

It was noteworthy that the majority of our of female patients had low educational attainment (63%) and no income (78%). Our participants were derived from a public restructured hospital with subsidised healthcare, suffice to say, the demographics of our study population reflect the exact community of women most in need of our educational efforts to help manage heart disease. In an observational cohort study of 5632 patients looking at educational level and CV disease prevention, patients with lower educational level were more often females, had more co-morbidities, more uncontrolled risk factors and a lower health related quality of life [32]. A heart health intervention programme delivered through our women’s heart clinic would potentially be influential in reducing the burden of heart disease in women, especially when directed at this target population.

## Limitations

In this pilot study, the small numbers, short duration of follow up and low event rates were the main limitations in detecting a good effect size of our intervention programme. Future research should also include identification of barriers in secondary prevention of CV disease, more targeted efforts in providing education and motivation for therapeutic lifestyle modification.

Also, in this study design, we recruited all female patients seen in the cardiac clinic, without taking into account the duration of follow up with their regular cardiologists. It would be more insightful to recruit and randomise only patients newly diagnosed to have CV disease at the cardiac clinics, so that there is direct comparison of both programmes right at the onset. This way, the time to treatment goals could also be measured, and there might also be more discernible changes in attitudes, knowledge and practices as well as quality of life in a short time span of 1 year follow up.

While there are accumulating evidence of sex disparities in CV management, it will be an over-simplification to propose that all women with CV disease would benefit from a sex-specific CV care. In this study, we recruited women from the general cardiology clinics with a broad heterogeneous group of CV diagnoses. The advantages of a sex-tailored CV programme might be better elucidated, if we had limited enrolment to those with cardiac conditions which have a predilection for women, or those associated with poorer outcomes in women. These could include: 1) young women with acute coronary syndrome below 60 years old, especially if they have diabetes, 2) those with angina or documented ischemia despite non-obstructive coronary arteries, 3) women with high CV risk scores or poor risk factor control, 4) women with recurrent presentations for heart failure symptoms, 5) women with CV disease in the peri-menopausal age group and those in the reproductive age group.

## Conclusion

This prospective randomised clinical trial is the first of its kind evaluating a new model of care for women with heart disease. We demonstrated the feasibility of a women’s heart health programme integrating disease management and education and lifestyle modification with a cultured-centred approach, but we were unable to demonstrate significant differences in quality of life, CV events or knowledge of CV disease. The potential to improve CV risk factor control and outcomes needs to be studied in a larger prospective trial with longer follow up. Continued evaluation of sex-specific heart programs is needed to provide supportive evidence for this important endeavor.

## Abbreviations

AF: Atrial fibrillation
CV: Cardiovascular
gSEM: Generalized structural equation model
IHD: Ischemic heart disease
MCS: Mental component score
NUHCS: National University Heart Center, Singapore
PCS: Physical component score
WHH: Women’s heart health
